# ABL Tyrosine Kinase Inhibition Variable Effects on the Invasive Properties of Different Triple Negative Breast Cancer Cell Lines

**DOI:** 10.1371/journal.pone.0118854

**Published:** 2015-03-24

**Authors:** Clément Chevalier, Aude Cannet, Simon Descamps, Audrey Sirvent, Valérie Simon, Serge Roche, Christine Benistant

**Affiliations:** 1 Centre de Recherche de Biochimie Macromoléculaire CNRS UMR5237, University of Montpellier, Montpellier, 34000, France; 2 Centre de Biochimie Structurale, CNRS UMR 5048, INSERM UMR 1054, University of Montpellier, Montpellier, 34090, France; University of Toledo, UNITED STATES

## Abstract

The non-receptor tyrosine kinase ABL drives myeloid progenitor expansion in human chronic myeloid leukemia. ABL inhibition by the tyrosine kinase inhibitor nilotinib is a first-line treatment for this disease. Recently, ABL has also been implicated in the transforming properties of solid tumors, including triple negative (TN) breast cancer. TN breast cancers are highly metastatic and several cell lines derived from these tumors display high invasive activity *in vitro*. This feature is associated with the activation of actin-rich membrane structures called invadopodia that promote extracellular matrix degradation. Here, we investigated nilotinib effect on the invasive and migratory properties of different TN breast cancer cell lines. Nilotinib decreased both matrix degradation and invasion in the TN breast cancer cell lines MDA-MB 231 and MDA-MB 468. However, and unexpectedly, nilotinib increased by two-fold the invasive properties of the TN breast cancer cell line BT-549 and of Src-transformed fibroblasts. Both display much higher levels of ABL kinase activity compared to MDA-MB 231. Similar effects were obtained by siRNA-mediated down-regulation of *ABL* expression, confirming ABL central role in this process. ABL anti-tumor effect in BT-549 cells and Src-transformed fibroblasts was not dependent on EGF secretion, as recently reported in neck and squamous carcinoma cells. Rather, we identified the TRIO-RAC1 axis as an important downstream element of ABL activity in these cancer cells. In conclusion, the observation that TN breast cancer cell lines respond differently to ABL inhibitors could have implications for future therapies.

## Introduction

Breast tumors are very heterogeneous and can be classified in three main groups based on their molecular profile: luminal cancers that express both estrogen and progesterone receptors; HER2-positive cancers that express the tyrosine kinase receptor ERBB2; and triple negative (TN) cancers in which none of these receptors is detected. TN breast cancers are the most aggressive and have the worst prognosis due to the lack of specific therapies [1]. Therefore, much research is currently focused on identifying the signaling pathways promoting TN cancer metastatic progression.

Tyrosine kinases (TK) have recently emerged as potentially important determinants of this process. We [2] and others [3, 4, 5] found that ABL kinases could play a role in TN breast cancer development and progression. ABL kinases form a family of ubiquitously expressed non-receptor TKs that include two members: ABL and ARG (Abl-related gene). Both proteins localize to the cell membrane, the actin cytoskeleton and the cytosol, and ABL is also present in the nucleus. Their modular organization and their mechanisms of regulation are very similar to that of non-receptor TKs of the Src family (SFK). However, in ABL and ARG, the regulatory C-terminal sequence of Src is replaced by a large sequence that includes F- and G-actin binding domains, proline-rich domains and nuclear localization signals, and, in the case of ABL, a DNA binding sequence. Like most TKs, the ABL family comprises oncogenic forms that exhibit strict cytoplasmic localization and deregulated kinase activity. These include the retroviral oncoprotein v-Abl expressed by the Abelson murine leukemia virus and the human BCR-ABL fusion oncoprotein that is responsible for human chronic myeloid leukemia (CML) [6]. ABL is thus an important therapeutic target in CML and several small inhibitors that target the ATP binding pocket in the TK catalytic domain have been developed. Imatinib, the first clinically available ABL tyrosine kinase inhibitor (TKI), has rapidly become the first-line treatment for CML. However, the appearance of resistance or intolerance to imatinib has led to the development of second generation TKIs. For instance, nilotinib is 30 times more potent than imatinib and has now replaced imatinib as first-line treatment. Similarly, dasatinib is 300 times more potent than imatinib and is effective to overcome resistance to imatinib. However, dasatinib increased efficiency was obtained at the expense of specificity and this inhibitor is now considered to be a multi-tyrosine kinase inhibitor [7]. In the absence of oncogenic mutations, ABL kinases are activated downstream of growth factor receptors or SFKs and can mediate many cell responses, such as proliferation, migration, endocytosis, cell transformation and epithelial-mesenchymal transition [3, 4, 6]. Particularly, ABL kinases are important regulators of actin cytoskeleton remodeling during cancer cell migration and invasion [8]. Moreover, they are involved in invadopodia maturation by directly phosphorylating cortactin at Y421 and Y470 [9]. However, when activated in the nucleus, ABL kinases also exert negative proliferative functions and promote apoptosis, resulting in the inhibition of tumor progression. For instance, the adhesive ephrin type-B receptor 4 (EPHB4) inhibits MDA-MB 435 breast cancer cell invasion through an ABL-CrkII signaling pathway [10] and ABL activation can inhibit TGFβ oncogenic signaling in the murine breast cell line 4T1 [11]. In agreement, a recent report demonstrated that ABL kinases negatively regulate invadopodia function and cell invasion of head and neck squamous cell carcinoma, through inhibition of a heparin binding epidermal growth factor-like growth factor (HB-EGF) autocrine loop [12].

Besides the ABL kinases, important regulators of actin cytoskeleton remodeling include members of the p21 RHO family of small GTPases, such as CDC42, RHOA, RHOC and RAC. These GTPases oscillate between the GTP- and GDP-loaded states in function of a fine balance of guanine nucleotide exchange factors (GEF), GTPase activating proteins (GAP) and guanine nucleotide dissociation inhibitors (GDI) [13]. Recently, it has been shown that a signaling cascade composed of the GEF TRIO, the RHO GTPase RAC and its effector PAK1 induces invadopodia disassembly, an important event required for invadopodia turnover to promote cell invasion [14]. Interestingly, the ABL kinases can regulate RAC localization and activity to control cell spreading [15] and cooperate with TRIO in regulating the actin cytoskeleton in neuronal growth cones [16].

Here, we investigated the effect of various ABL TKIs on the migratory and invasive properties of different TN breast cancer cell lines. We found that nilotinib inhibits the invasive properties of MDA-MB 231 and MDA-MB 468 cells, while it increases the invasiveness of BT-549 cells. Nilotinib also promoted invasiveness of Src-transformed fibroblasts. This result indicates that TN breast cancer cell lines respond differently to ABL inhibitors. We also found that the GEF TRIO and the GTPase RAC1 are potential downstream elements of ABL kinase negative regulation of invadopodia in TN breast cancer cells.

## Materials and Methods

### Reagents, antibodies and constructs

Antibodies against ABL (clone 8E9 and AB3) were from BD Bioscience and Calbiochem, respectively. Antibodies against CST1, p-Tyr (4G10) and tubulin were homemade or from P. Mangeat and N. Morin (CRBM, Montpellier France), respectively. Anti-TRIO antibodies were as described in [17]. Alexa 598-phalloidin and Oregon Green 488–conjugated gelatin were from Molecular Probes. Imatinib, nilotinib and dasatinib were from LC laboratories, SU6656 and NCS23766 from Calbiochem. C3 exoenzyme (*Clostridium botulinum*), enolase (rabbit muscle), poly-D lysine and EGF were from Sigma. ITX3 [18] was from P. Fort (CRBM, Montpellier, France). SiRNA against human *ABL* were from Qiagen. Lipofectamine 2000 was from Invitrogen. The shTrio1 and shTrio2 shRNAs against mouse and human *TRIO* were described in [19]. mCherry-TRIO and mCherry-GEFD1 were described in [20]. Matrigel and Boyden chambers were from BD Bioscience. Immobilon membranes were from Millipore. Protein-G agarose, gP^32^ ATP and ECL were from Amersham,

### Cell culture, transfections, retroviral infections and treatments

MDA-MB 231, MDA-MB 468 and BT-549 cells from ATCC hosted at IRCM Montpellier, France and NIH-3T3 SrcY527F cells (3T3 SrcY527F) (a gift by S. Courtneidge, Burnham Institute, San Francisco, USA and described in [21]) were cultured in Dulbecco’s modified Eagle’s medium (DMEM) supplemented with 10% fetal calf serum (FCS), glutamine and antibiotics (penicillin and streptomycin) at 37°C in a humidified 5% CO_2_ atmosphere. Transient transfections were performed with Lipofectamine 2000 according to the manufacturer’s instructions. Cells were transfected 48 h before imaging or lysis. Retroviral infection procedures were as described in [22] and stable cell lines were obtained by selection with 200μg/ml hygromycin. For inhibitor assays, cells were seeded on gelatin (see below), briefly spun down and treated with 5 μM SU6656, 5 μM imatinib, 10–100 nM nilotinib, 100nM dasatinib, 2μg/ml C3, 100μM NCS23766 or 25μM ITX3 for 3 h except for EGF experiments. In this case, cells were serum-starved (0.5% serum) overnight prior to seeding and were then stimulated with 200mg/ml EGF for 3h.

### Gelatin degradation assay and invadopodia imaging

Gelatin degradation assays were performed as described previously [23]. Coverslips were ethanol-washed and coated with 50 μg/ml poly-D-lysine for 15 min, washed with PBS and cross-linked with 0.5% glutaraldehyde for 15 min. Coverslips were then inverted on a 20 μl drop of 1 mg/ml Oregon-Green 488–conjugated gelatin (Molecular Probes) for 20 min. After washing with PBS, coverslips were quenched with 5 mg/ml sodium borohydride for 5 min followed by washes with PBS. Finally, they were transferred in complete growth medium for 30 min-1 h before use. Cells were seeded and cultured on cross-linked gelatin for 3h in the absence (vehicle) or presence of inhibitors and then fixed for immunofluorescence studies. Wide-field imaging was done using Zeiss AxioimagerZ1 or Zeiss AxioimagerZ2 upright microscopes with Zeiss 40 X EC Plan NeofluaR 1.3 oil DIC and Zeiss 63X Plan-Apochromat 1.4 oil objectives. Image acquisition and quantification of gelatin degradation areas were carried out using Metamorph (Molecular Devices, Inc.).

### Migration and invasion assays

Cell migration and invasion assays were performed in Boyden chambers. For migration assay, 20000 cells were added on the upper chamber containing medium with 0.5% FCS, while the lower chamber contained 10% FCS. For invasion assay, 50000 cells were added on the upper chamber containing 100 μl of 750 μg/ml Matrigel. Cells were fixed after 45 min (migration) or 24–48 h (invasion) in 3.7% paraformaldehyde solution containing 0.1% Triton X100 and 0.1% Hoechst 33342. Whole well images were acquired using a Zeiss Axiovert 200M or Leica DMIRE2 inverted microscope and a 10X EC Plan Neofluar 0.3 PH1 objective. Nuclei were counted in whole wells using the Metamorph software (Molecular Devices, Inc.).

### Biochemical assays

Immunoprecipitation experiments, western blotting and kinase assays were performed as described in [22, 2]. Briefly, cells were rinsed twice in PBS and scraped in 2X RIPA lysis buffer or in lysis buffer containing 1% Triton X-100 or 1% NP40, 10mM Tris-HCl (pH 7.5), 150 mM NaCl, 5 mM EDTA, 75U/ml aprotinin, and 1 mM vanadate. Immunoprecipitations were done using 800μg-1mg protein extracts and specific antibodies. 20 to 50 μg proteins were used as whole cell lysate (WCL). Immunoprecipitates and WCL were separated on 5–10% SDS-PAGE gels and transferred to Immobilon membranes. Detection was performed using the ECL System.

## Results

### Variable effects of ABL kinase inhibition on the invasive properties of TN breast cancer cell lines

**Fig 1:**
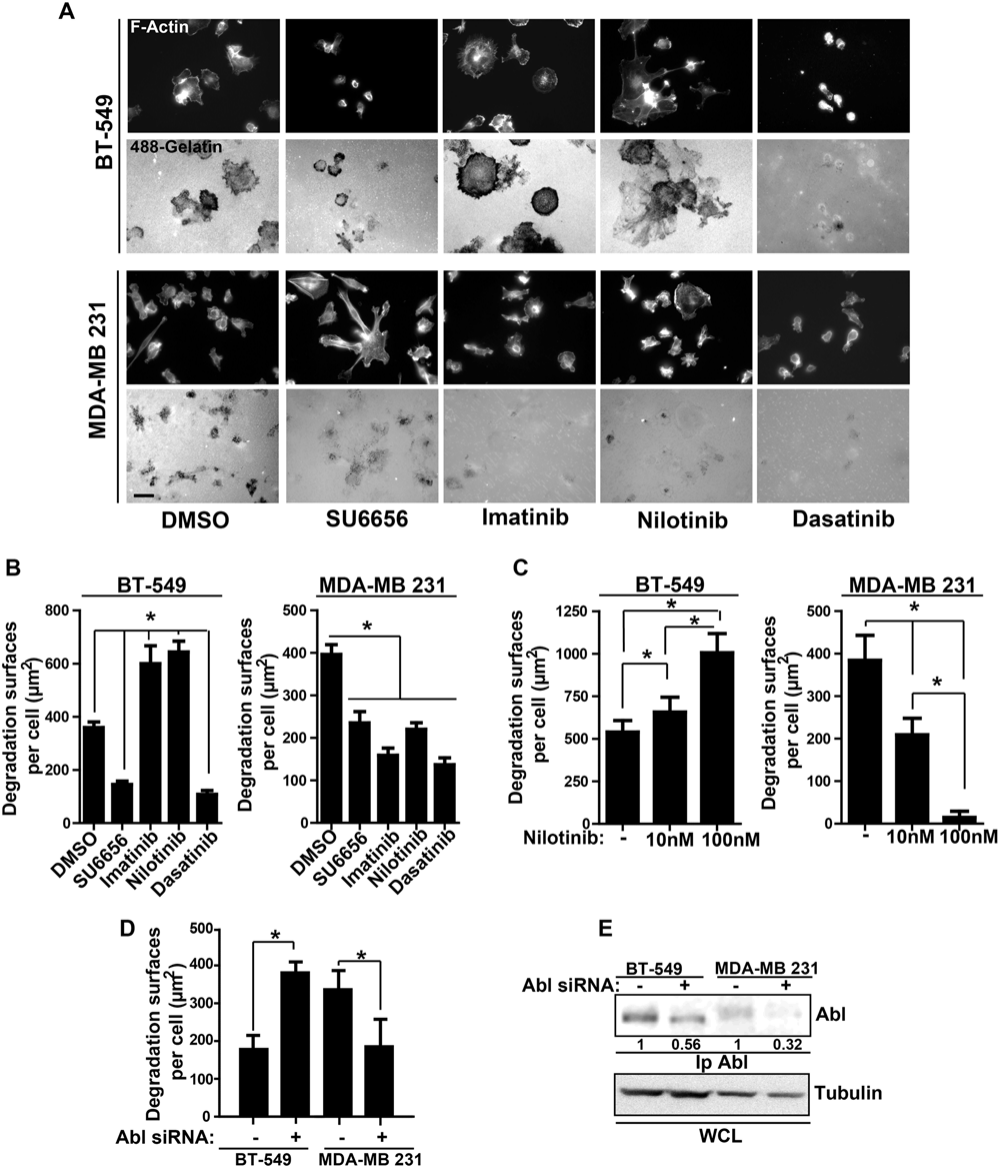
Variable outcomes of ABL kinase inhibition on the invasive properties of TN breast cancer cells. **(A)** BT-549 and MDA-MB 231 cells were seeded on Oregon Green 488 gelatin-coated coverslips and treated with DMSO, 5μM SU6656, 5μM imatinib, (10–100) nM nilotinib or 100nM dasatinib for 3h and then fixed for immunofluorescence studies. The actin cytoskeleton morphology was visualized by labeling F-actin with Alexa 598-conjugated phalloidin and the cell degradation activity was assessed based on the appearance of black spots, due to gelatin degradation, in the green fluorescent background of the gelatin matrix. Scale bar: 20μm. **(B)** Quantification of the matrix degradation area per cell (in μm^2^; mean ± SEM of more than 100 cells/condition from three different experiments). *p<0.05. **(C)** BT-549 and MDA-MB 231 cells were seeded on Oregon Green 488 gelatin-coated coverslips and treated with DMSO or two different concentrations of nilotinib (10nM and 100nM) for 3h and then fixed for immunofluorescence analysis to quantify the degradation area per cell (in μm^2^; mean ± SEM). *p<0.05 **(D)** BT-549 cells were transfected twice at an interval of 24h control siRNA (Ctrl) or *ABL* siRNA. After 48h, cells were plated on Oregon Green 488 gelatin-coated coverslips for 3h. After fixation, the degradation area per cell (in μm^2^; mean ± SEM) was quantified. *p<0.05, NS: non-significant. **(E)** BT-549 and MDA-MB 231 cells transfected twice with control (Ctrl) or *ABL* siRNAs as in (D) were lysed and immunoprecipitated with an anti-ABL antibody. Densitometry quantification normalized to tubulin is shown.

**Fig 2:**
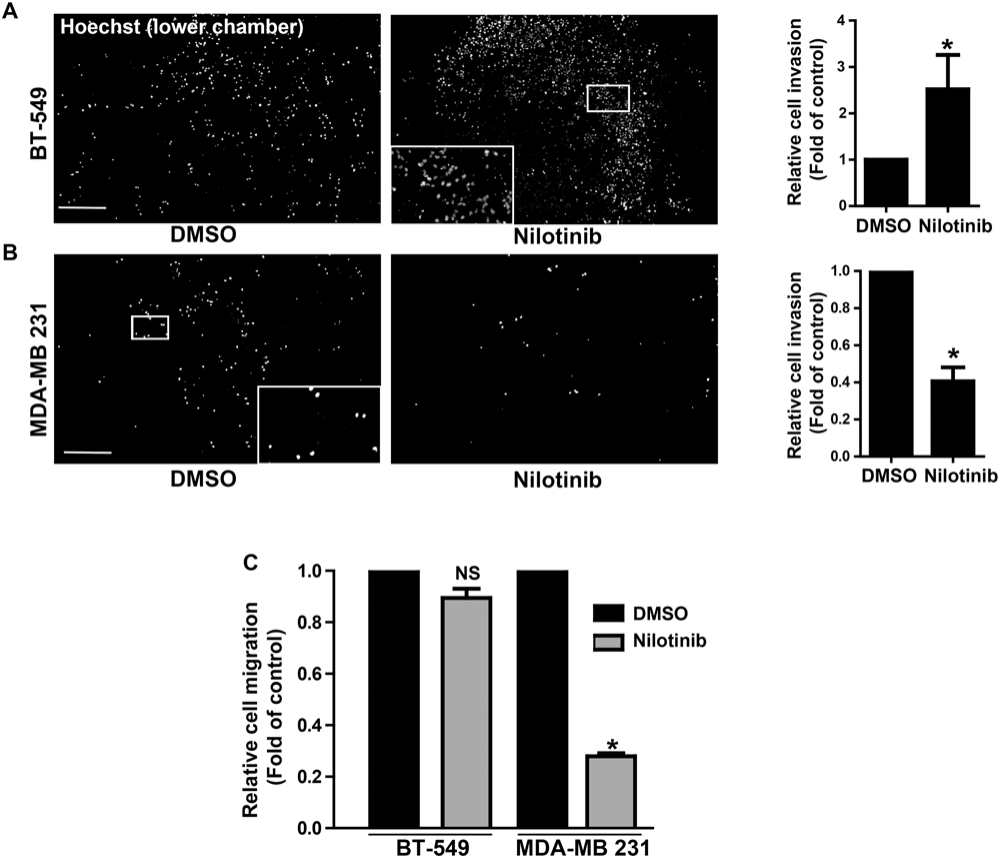
Nilotinib effect on cancer cell invasion and migration. **(A, B)** BT-549 cells and MDA—MB 231 were seeded in the Matrigel-coated upper compartment of Boyden chambers and incubated with DMSO or 100nM nilotinib for 24h before fixation. Cell nuclei in the lower chamber were stained with Hoechst (representative images in left panels, scale bar: 500μm. Insets show higher magnification of the boxed region) to quantify in whole well the number of cells that migrated from the top chamber through the Matrigel-coated filter pores. Shown is the cell invasion (mean ± SD, n = 3 experiments, right panels) relative to control (DMSO); *p<0.05 compared to control cells. **(C)** Cells were seeded in the upper compartment of Boyden chambers and incubated with DMSO or 100nM nilotinib for 45 min; quantification (mean ± SD, n = 3) of cells that migrated through the uncoated filter was as in (A); *p<0.05, NS: non-significant compared to DMSO treated cells.

**Fig 3:**
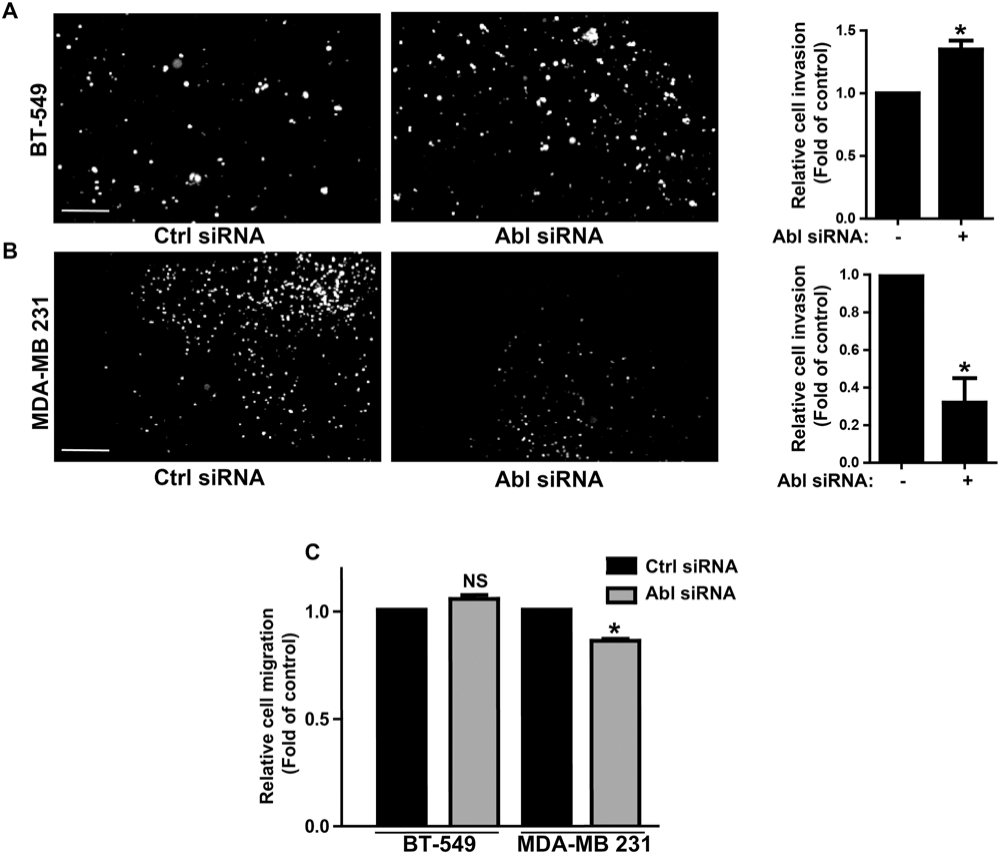
Abl downregulation effect on cancer cell invasion and migration. **(A, B)** BT-549 cells and MDA—MB 231 were transfected twice with control (Ctrl) and Abl siRNAs and were seeded in the Matrigel-coated upper compartment of Boyden chambers and incubated for 24h before fixation. Cell nuclei in the lower chamber were stained with Hoechst (representative images in left panels. Scale bar: 250μm) to quantify in whole well the number of cells that migrated from the top chamber through the Matrigel-coated filter pores. Shown is the cell invasion (mean ± SD, n = 3 experiments, right panels) relative to ctrl siRNA; *p<0.05 compared to control cells. **(C)** Ctrl and Abl siRNA transfected cells were seeded in the upper compartment of Boyden chambers and incubated for 45 min. Quantification (mean ± SD, n = 3) of cells that migrated through the uncoated filter was as in (A); *p<0.05, NS: non-significant compared to Ctrl siRNA.

We first determined whether ABL kinase inhibitors currently used in the clinic affect the invasive properties of three TN breast cancer cell lines (MDA-MB 231, BT-549, and MDA-MB 468) and of transformed cells the reference model cell line 3T3 SrcY527F. To this aim, we plated cells on Oregon Green 488-conjugated gelatin-coated coverslips and then assessed invadopodia activity in each cell line after incubation with DMSO (negative control), imatinib, nilotinib, dasatinib or the SRC-like inhibitor SU6656 (positive control) by quantifying the dark areas over the green background. SU6656 and dasatinib inhibited the invasive activity of all cell lines, whereas imatinib and nilotinib had an inhibitory effect only in MDA-MB 231 and MDA-MB 468 cells (Fig. 1A, B and S1 Fig. A, B). Unexpectedly, both inhibitors increased by 2-fold gelatin degradation in BT-549 (Fig. 1A, B) and 3T3 SrcY527F cells (S1 Fig. A, B). Both effects were dose-dependent (Fig. 1C). Nilotinib-mediated degradation increase was associated with profound remodeling of the actin cytoskeleton as indicated by the appearance of increased number of F-actin punctae, indicative of invadopodia in BT-549 (Fig. 1A) and many podosome rosettes in 3T3 SrcY527F cells (S1 Fig. A). These data suggest that imatinib and nilotinib increase matrix degradation by inducing invadopodia, in agreement with a recent report showing that the ABL kinases negatively regulate invadopodia/podosomes in 3T3 SrcY527F cells [12]. As imatinib and nilotinib can also target other TKs [24], we confirmed the involvement of ABL in this process by down-regulating *ABL* expression using specific siRNAs. ABL depletion resulted in increased matrix degradation in BT-549 cells and reduced degradation in MDA-MB 231 cells, like upon incubation with the inhibitors (Fig. 1D, E). This confirmed the central role of ABL in this cellular process, although a contribution of ARG cannot be excluded. In breast cancer, the ability of cells to form invadopodia is tightly linked to their invasive potential. Accordingly, incubation with nilotinib increased by 2.5-fold invasion of BT-549 (Fig. 2A) and 3T3 SrcY527F cells (S2 Fig. A) through Matrigel, whereas it reduced by 2-fold invasion of MDA-MB 231 cells compared to control cells (DMSO) (Fig. 2B). Conversely, nilotinib strongly reduced migration of MDA-MB 231 cells, but not of BT-549 (Fig. 2C) and 3T3 SrcY527F cells (S2 Fig. B), suggesting that ABL does not regulate tumor cell motility on its own. This was further confirmed by *ABL* expression down regulation using specific siRNAs (Fig. 3A, B and C).

### The HB-EGF autocrine loop is not involved in ABL negative regulation of BT-549 cell invasion

**Fig 4:**
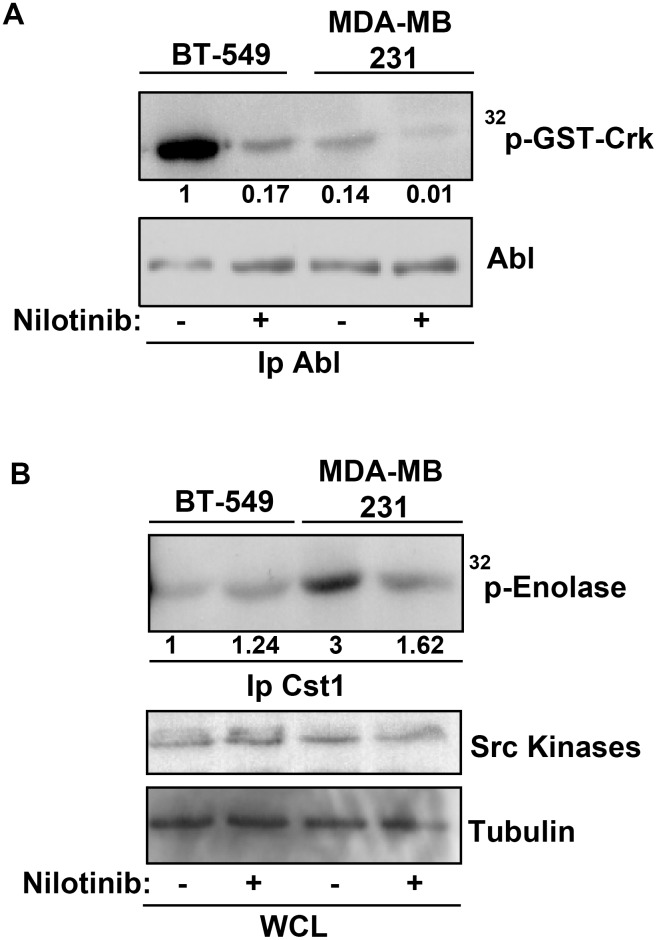
Nilotinib effect on ABL and SRC tyrosine kinase activity. **(A)** Triton X-100 lysates of BT-549 and MDA-MB 231 cells incubated with DMSO (-) or 100nM nilotinib (+) were used for *in vitro* kinase assays to test ABL kinase activity. Immunoprecipitates were incubated with GST-Crk as a substrate and P^32^-labeled ATP. P^32^ incorporation by GST-Crk was detected by autoradiography. The Abl content of immunoprecipitates and densitometry quantification normalized to Abl are also shown. **(B)** Triton X-100 lysates of BT-549 and MDA-MB 231 cells incubated with DMSO (-) or 100nM nilotinib (+) were immunoprecipitated with an anti-CST1 antibody that recognizes SRC, FYN and YES. *In vitro* kinase assay to test SRC activity was performed using enolase as substrate and P^32^-labeled ATP. P^32^ incorporation by enolase was detected by autoradiography. Src and tubulin are used as loading control. Densitometry quantification normalized to tubulin is shown.

**Fig 5:**
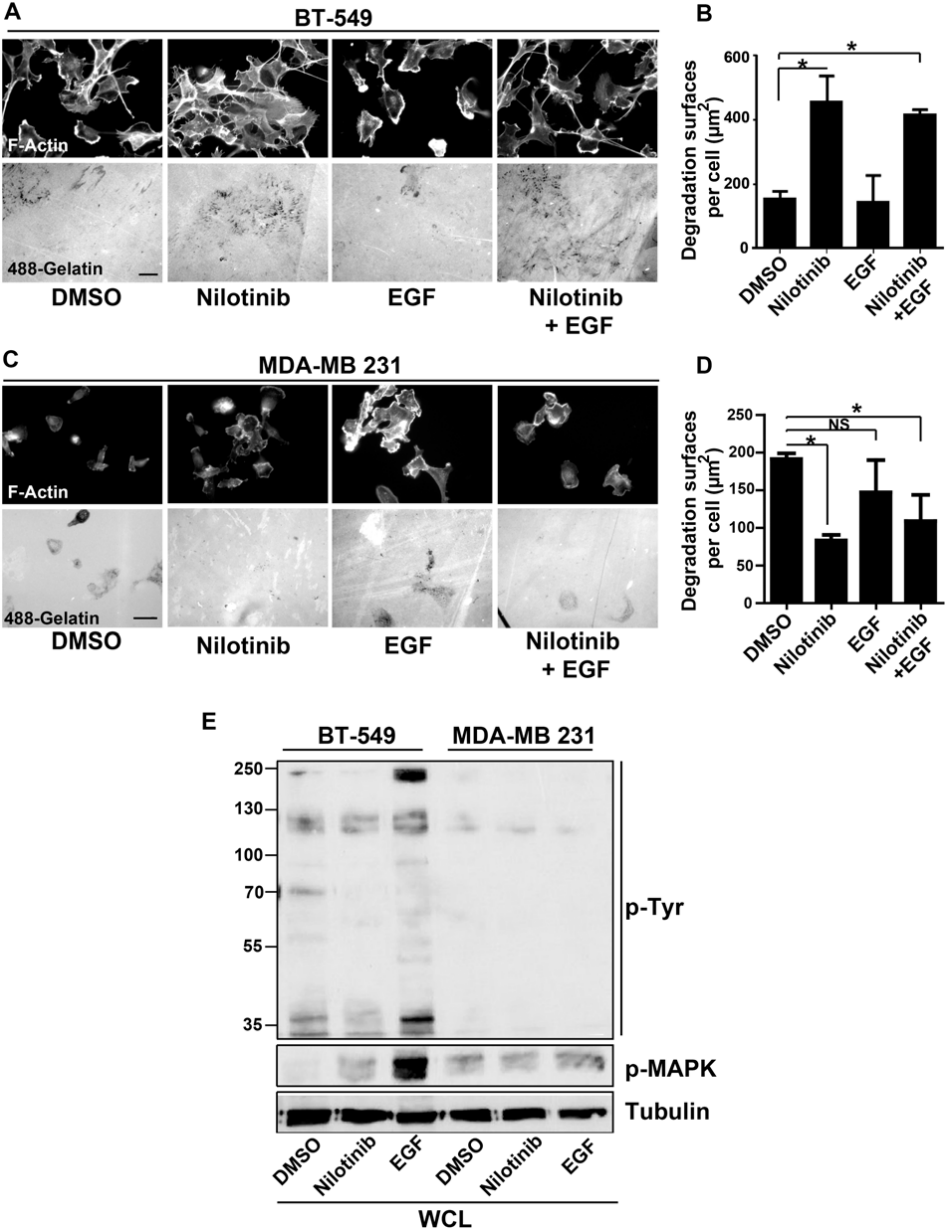
The HB-EGF autocrine loop is not regulated by the ABL kinase in BT-549 cells. BT-549 cells **(A, B)** and MDA-MB 231 **(C, D)** were serum-starved (0.5% serum) overnight, then plated on Oregon Green 488 gelatin and incubated with DMSO, 100nM nilotinib, 200ng/ml EGF or EGF+nilotinb for 3h. After fixation, actin cytoskeleton morphology and gelatin degradation by cells were analyzed as in Fig. 1A. Scale bars: 20μm. Results are the mean ± SEM, *p<0.05 compared to DMSO-treated cells. **(E)** Tyrosine phosphorylation profile and phosphorylated MAPK levels in Triton X-100 lysates from BT-549 and MDA-MB 231 cells incubated with DMSO, 100nM nilotinib or 200ng/ml EGF were assayed as described in Fig. 4. Tubulin was used as loading control. Molecular weights (kDa) are indicated on the left.

We next investigated the underlying mechanism of ABL anti-invasive activity in BT-549 and 3T3 SrcY527F cells. These two cell lines display the highest ABL kinase activity levels among the cells used in this study (Fig. 4A and S2 Fig. D). Nilotinib did not have any effect on SFK activity in BT-549 cells (Fig. 4B) and 3T3 SrcY527F (S2 Fig. E), suggesting that ABL kinase inhibition did not have any indirect effect on SFK activity, differently from what reported in [12]. Accordingly, nilotinib did not have any appreciable effect on the global tyrosine phosphorylation level in BT549 (Fig. 5E) and 3T3 SrcY527F cells (S2 Fig. C). These findings exclude a potential negative ABL feedback loop towards SFK activity. The situation was different in MDA-MB 231 where Abl inhibition by nilotinib reduced SFK activity and this might explain why Abl inhibition strongly impacted on matrix degradation in this case (Fig. 4B). A recent study described an HB-EGF-dependent autocrine loop involved in invadopodia activity and regulated by ABL kinase activities [12]. As this ABL-dependent feedback loop was observed only in serum-starved conditions, we quantified gelatin degradation after serum starvation and incubation, or not, with EGF and/or nilotinib in Src-transformed fibroblasts. Gelatin degradation after serum starvation was similarly increased by incubation with EGF and/or nilotinib (S3 Fig. A, B), as described in [12]. Conversely, EGF had no effect on BT-549 cells (Fig. 5A, B), although the EGF receptor was well expressed and activated as indicated by the increased levels of tyrosine phosphorylation and phosphorylated MAPKs upon incubation with EGF (Fig. 5E). In MDA MB231 cells, that expressed low amount of the receptor (Fig. 5E) we observed no significant effect of EGF on gelatin degradation (Fig. 5C, D) and as described in [12].

### RAC1 and TRIO may act downstream of ABL kinases to control cell invasion in BT-549 cells and Src-transformed fibroblasts

**Fig 6:**
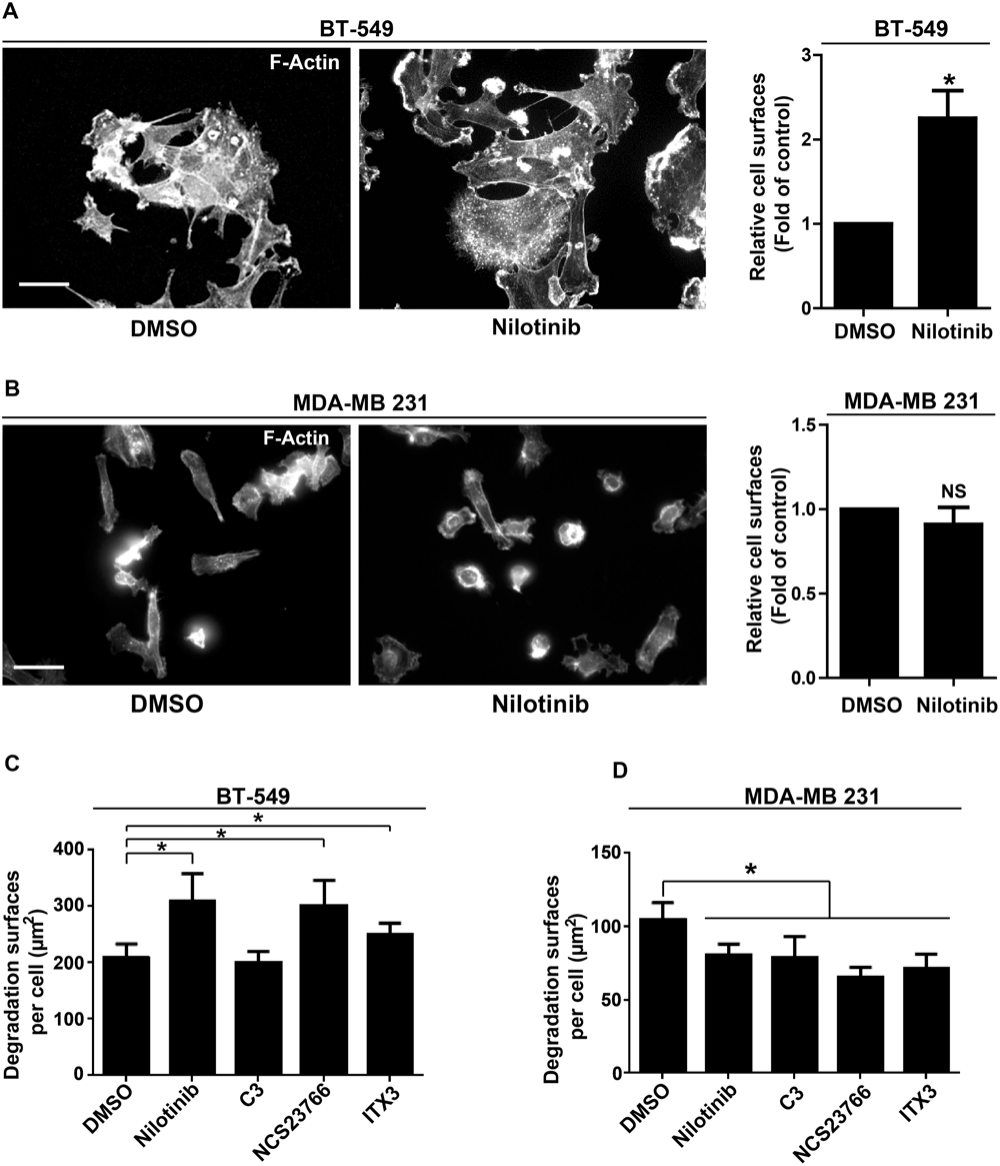
Effect of nilotinib and RHO inhibitors on cell area and matrix degradation. **(A, B)** Effect of nilotinib on BT-549 and MDA-MB 231 cell area. The area of 100 BT-549 (A) and MDA-MB 231 (B) cells plated on Oregon Green 488 gelatin was measured after incubation with DMSO or 100nM nilotinib and F-actin staining. Results are the mean ± SEM relative to control. *p<0.05 compared to DMSO-treated cells. Scale bar: 20μm. **(C, D)** Degradation area of 100 BT-549 (C) and MDA-MB 231 (D) cells incubated with DMSO, 100nM nilotinib, 2μg/ml C3, 100μM NSC23766 or 25μM ITX3 for 3h were measured as described in Fig. 1A. Results are the mean ± SEM, *p<0.05 compared to DMSO-treated cells.

**Fig 7:**
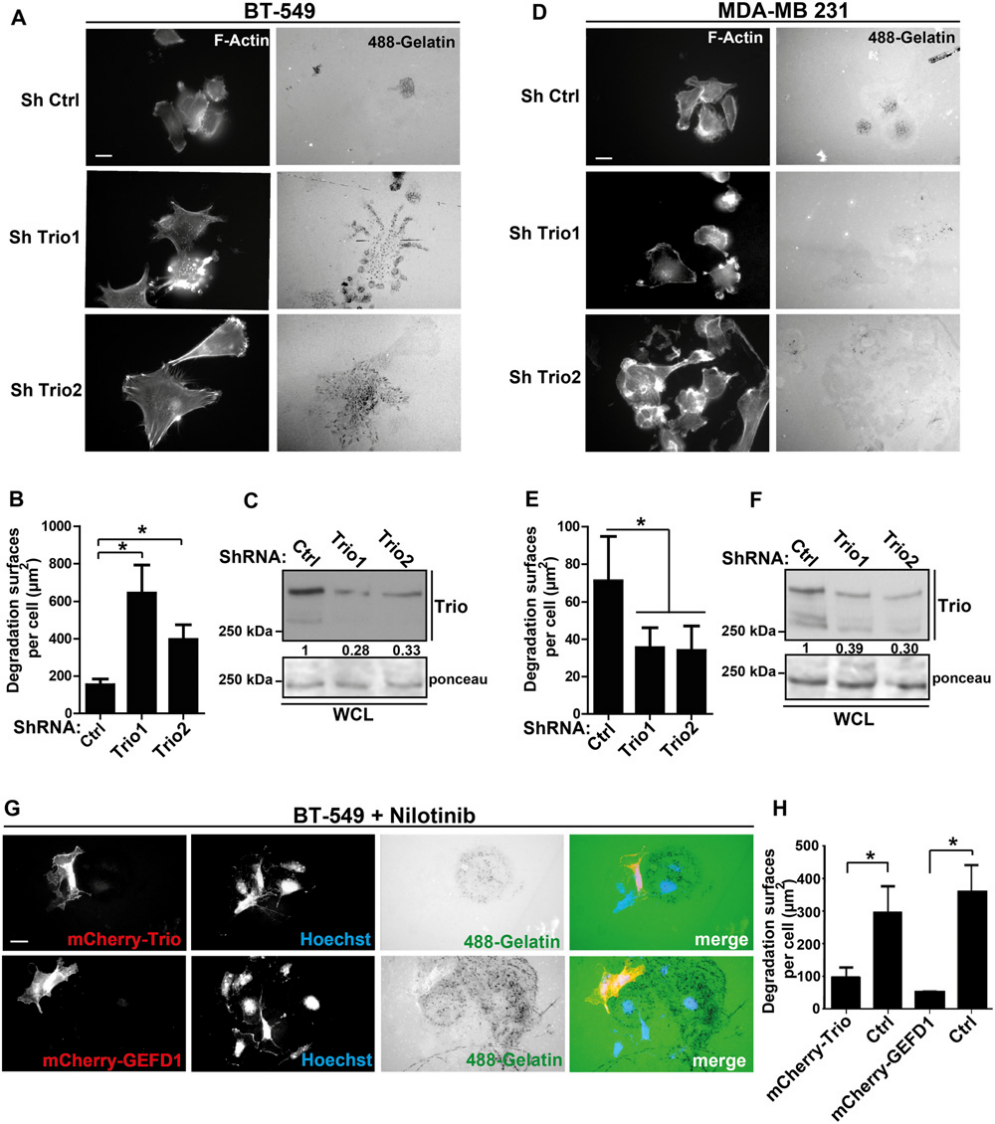
Involvement of the TRIO/RAC1 axis downstream of the ABL kinases in the regulation of invadopodia. **(A, B)** BT-549 cells were infected with retroviruses expressing control (ShCtl) or anti-*TRIO* shRNAs (ShTrio1 and ShTrio2) and then plated on Oregon Green 488 gelatin. After fixation, actin cytoskeleton morphology and gelatin degradation by cells were analyzed as described in Fig. 1A. **(C)** TRIO expression in lysates from BT-549 cells infected with control shRNA, ShTrio1 or ShTrio2. Densitometry quantification normalized to ponceau is shown. **(D, E and F)** Actin cytoskeleton morphology and gelatin degradation by MDA-MB 231 cells infected with control shRNA, ShTrio1 or ShTrio2. **(G)** BT-549 cells were transfected with plasmids encoding mCherry-Trio or the GEF domain 1 (D1) of TRIO (mCherry-GEFD1). After 48h cells were plated on Oregon Green 488 gelatin and incubated with 100nM nilotinib. **(H)** The degradation area in transfected BT-549 cells was quantified as in Fig. 1B. *p<0.05. Scale bar: 20μm.

Incubation with nilotinib also increased the area of BT-549 (Fig. 6A) and 3T3 SrcY527F cells (S4 Fig. A) compared to cells treated with DMSO alone or to that observed in MDA-MB 231 cells (Fig. 6B). This observation is consistent with a previous report showing the increase of *Abl-/-* fibroblast area upon spreading and implicating the small GTPase RAC1 [15]. We thus investigated whether RAC1 could be involved in ABL anti-invasive activity. Like with nilotinib, incubation with NSC 23766 (a RAC1 inhibitor) also increased the cell area (not shown) and gelatin degradation in BT-549 and 3T3 SrcY527F cells (Fig. 6C, D and S4 Fig. B) but not in MDA-MB 231 cells (Fig. 6D). This effect was RAC-specific because the RHO GTPase inhibitor C3 did not have any effect (Fig. 6C and S4 Fig. B). To determine how RAC affects ABL anti-invasive signaling, we first investigated the role of the GEF TRIO because it controls RAC1 activity in invadopodia disassembly [14]. TRIO inhibition by sub-optimal doses (due to toxicity) of the pharmacological inhibitor ITX3 [18] significantly increased gelatin degradation by BT-549 and 3T3 SrcY527F cells compared to cells incubated with DMSO (Fig. 6C, D and S4 Fig. B). We further confirmed TRIO role by showing that *TRIO* down-regulation with two different shRNAs (shTrio1 and shTrio2) increased gelatin degradation by BT-549 (Fig. 7A, B and C) and 3T3 SrcY527F cells (S4 Fig. C, D and E), while it reduced gelatin degradation by MDA-MB 231 cells (Fig. 7D, E and F). Moreover, *TRIO* down-regulation strongly promoted the formation of podosome rosettes in 3T3 SrcY527F cells (S4 Fig. C). These findings suggest that ABL anti-invasive activity is mediated through a TRIO/RAC signaling pathway. To confirm this hypothesis, we investigated whether constitutive RAC1 activation could bypass nilotinib inhibition of ABL anti-invasive activity in 3T3 SrcY527F and BT-549 cells. Indeed, after incubation with nilotinib, the degradation areas of BT-549 cells transfected with mCherry-Trio were significantly smaller than in neighboring non-transfected cells (Ctrl) (Fig. 7G, H). Similar results were obtained in cells transfected with mCherry-GEFD1, a construct that includes only the RAC1-specific GEF domain of TRIO, demonstrating that this effect is specifically linked to RAC activation (Fig. 7G, H). Altogether, these results suggest that in BT-549 and 3T3 SrcY527F cells, ABL anti-invasive activity is mediated via a TRIO/RAC signaling pathway.

## Discussion

It would be tempting to extend the application of ABL-targeting therapies to other cancers given their success in CML. However, this study is in agreement with a growing list of reports showing contrasting roles of the ABL kinases in solid tumors [11, 12]. We found that ABL kinase inhibition by nilotinib or imatinib had opposite effects in BT-549 cells and MDA-MB 231 or MDA-MB 468 cells. The effect in BT-549 cells is similar to that in the mouse 4T1 breast cancer cell line, which is considered to be equivalent to human TN breast cancer cell lines in some reports [25]. How to explain the different outcomes of ABL inhibition in these different TN breast cancer cell lines? A recent study on the PKCα-PI3K signaling axis in the regulation of cell invasion retained our attention [26]. The authors demonstrated that PKCα has opposite effects on invadopodia formation, depending on the cell PI3K status: loss of PKCα inhibited invadopodia formation in cells harboring wild type PI3K signaling components, while it increased invadopodia formation in cells with deregulated PI3K signaling due to activating *PI3K* somatic mutations or *PTEN* inactivation. Particularly, the invasive activity of BT-549 cells, which do not express PTEN, was negatively regulated by PKCα [26]. On the other hand, PI3K/PTEN signaling is not mutated in MDA-MB 231 cells [27]. It would be interesting to further investigate whether and how ABL kinases might affect PI3K/PKCα signaling to control invadopodia formation.

We then found that TRIO and RAC1 might be downstream components of the signaling pathway whereby ABL negatively regulates cell invasion in BT-549 cells. This is in agreement with previous reports showing a link between ABL kinases and TRIO or RAC1 [15, 16] and with a recent study demonstrating a negative regulatory role of the TRIO-RAC1 axis on invadopodia activity of breast carcinoma cells [14]. What remains to be determined is how ABL is connected to TRIO. We could not identify TRIO as a direct ABL substrate because we did not detect tyrosine phosphorylation of TRIO in our assays (data not shown). While we cannot formally exclude TRIO regulation by tyrosine phosphorylation, ABL could regulate TRIO indirectly via PKCα- or PI3K-dependent mechanisms.

EGF is involved in this ABL negative function in head and neck squamous cell carcinoma. Conversely, we could not confirm such a mechanism in the TN breast cancer cell lines used in this study [12]. Other secreted factors could be involved, such as TGFβ, which is regulated by the ABL kinases in 4T1 breast cancer cells [11]. Netrin-1, which is specifically secreted by BT-549 cells, could also be an interesting candidate [28] because its receptor DCC can signal to TRIO [17] and is involved in invadopodia-driven basement membrane transmigration *in vivo* [29].

In conclusion, we found that, *in vitro*, ABL kinase role in the invasive activity of TN breast cancer cells varies in different cell lines. Consistent with our *in vitro* observation, dasatinib has limited activity, as single-agent, in unselected patients with metastatic breast cancer. Similarly, a recent phase II study in which patients were selected based on dasatinib predictive gene signatures defined in preclinical models did not report any satisfactory tumor response except in one patient [30]. Therefore, there is a clear need to identify novel and reliable markers for patient selection. Unraveling the molecular cause of the heterogeneous ABL effect on the invasive behavior of breast tumor cells might help identifying reliable markers for patient selection.

## Supporting Information

S1 Fig Variable outcomes of ABL kinase inhibition on the invasive properties of transformed cells and TN breast cancer cells. **(A)** 3T3 SrcY527F and MDA-MB 468 cells were seeded on Oregon Green 488 gelatin-coated coverslips and treated with DMSO, 5μM SU6656, 5μM imatinib, 100nM nilotinib or 100nM dasatinib for 3h and then fixed for immunofluorescence studies. The actin cytoskeleton morphology was visualized by labeling F-actin and the cell degradation activity was assessed as in Fig. 1. Scale bar: 20μm. **(B)** Quantification of the matrix degradation area per cell (in μm^2^; mean ± SEM of more than 100 cells/condition from three different experiments). *p<0.05.
(TIF)

S2 Fig Effect of Nilotinib on invasion, migration, Abl and Src kinases activities of 3T3 SrcY527F cells. **(A, B)** Cell invasion and migration studies were done as in Figs. 2 and 3, **(C-E)** Biochemical analyses were done as described in Figs. 4 and 5.
(TIF)

S3 Fig The HB-EGF autocrine loop is active in 3T3 Src Y527F. **(A, B)** 3T3 Src Y527F cells were serum-starved (0.5% serum) overnight, then plated on Oregon Green 488 gelatin and incubated with DMSO, 100nM nilotinib, 200ng/ml EGF or EGF+nilotinb for 3h. After fixation, actin cytoskeleton morphology and gelatin degradation by cells were analyzed as in Fig. 1A. Scale bars: 20μm
(TIF)

S4 Fig The TRIO/RAC1 axis regulates podosomes in 3T3 SrcY527F cells. **(A)** Effect of nilotinib on cell area. The area of 100 3T3 SrcY527F cells plated on Oregon Green 488 gelatin and incubated with DMSO or 100nM nilotinib was measured after F-actin labeling with Alexa 598-phalloidin. Results are the mean ± SEM relative to control. *p<0.05, compared to DMSO-treated cells. **(B)** The degradation area of 100 3T3 SrcY527F cells plated on Oregon Green 488 gelatin and incubated with DMSO, 100nM nilotinib, 2μg/ml C3, 100μM NSC 23766 or 25μM ITX3 for 3h was measured as described in Fig. 1A. Results are the mean ± SEM, *p<0.05 compared to DMSO treated-cells. **(C, D and E)** Effects of TRIO down-regulation with shTrio1 in 3T3 SrcY527F cells. (C) Representative examples of actin cytoskeleton morphology and matrix degradation in 3T3 SrcY527F cells infected with control (ShCtrl) or anti-Trio1 shRNAs. Scale bar: 20μm. (D) Quantification of matrix degradation. *p<0.05 compared to ShCtl cells. (E) Western blot showing TRIO expression in 3T3 SrcY527F cells infected with control (ShCtrl) or anti-Trio1 shRNAs. Densitometry quantification normalized to tubulin is shown.
(TIF)
