# Supplementary figures and images for: ABL Tyrosine Kinase Inhibition Variable Effects on the Invasive Properties of Different Triple Negative Breast Cancer Cell Lines
Source: PLoS One. 2015 Mar 24;10(3):e0118854. doi: 10.1371/journal.pone.0118854 (PMC4372365; doi:10.1371/journal.pone.0118854)

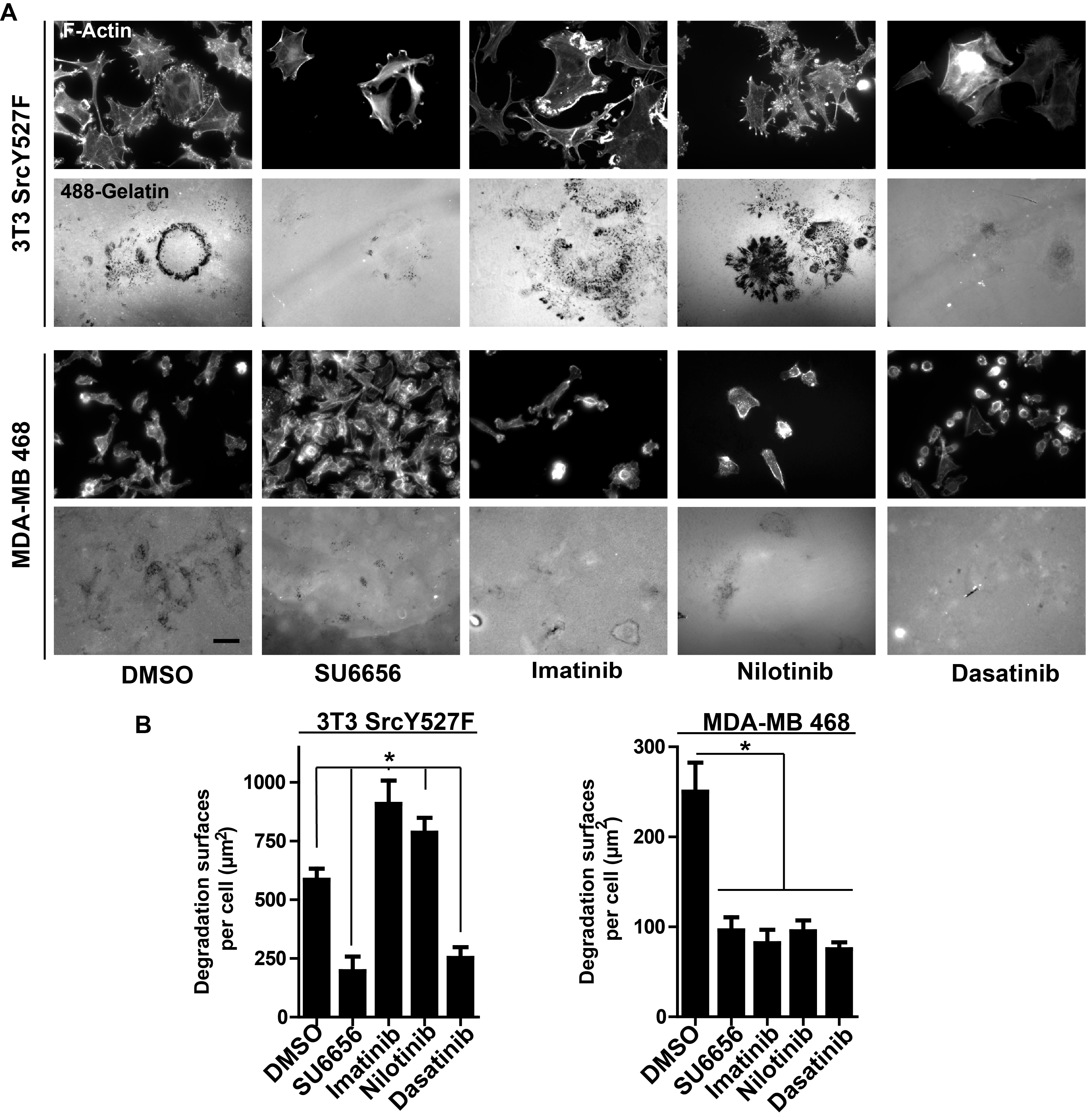

Supplement: S1 Fig — (A) 3T3 SrcY527F and MDA-MB 468 cells were seeded on Oregon Green 488 gelatin-coated coverslips and treated with DMSO, 5μM SU6656, 5μM imatinib, 100nM nilotinib or 100nM dasatinib for 3h and then fixed for immunofluorescence studies. The actin cytoskeleton morphology was visualized by labeling F-actin and the cell degradation activity was assessed as in Fig. 1. Scale bar: 20μm. (B) Quantification of the matrix degradation area per cell (in μm2; mean ± SEM of more than 100 cells/condition from three different experiments). *p<0.05. (TIF) [file pone.0118854.s001.tif]

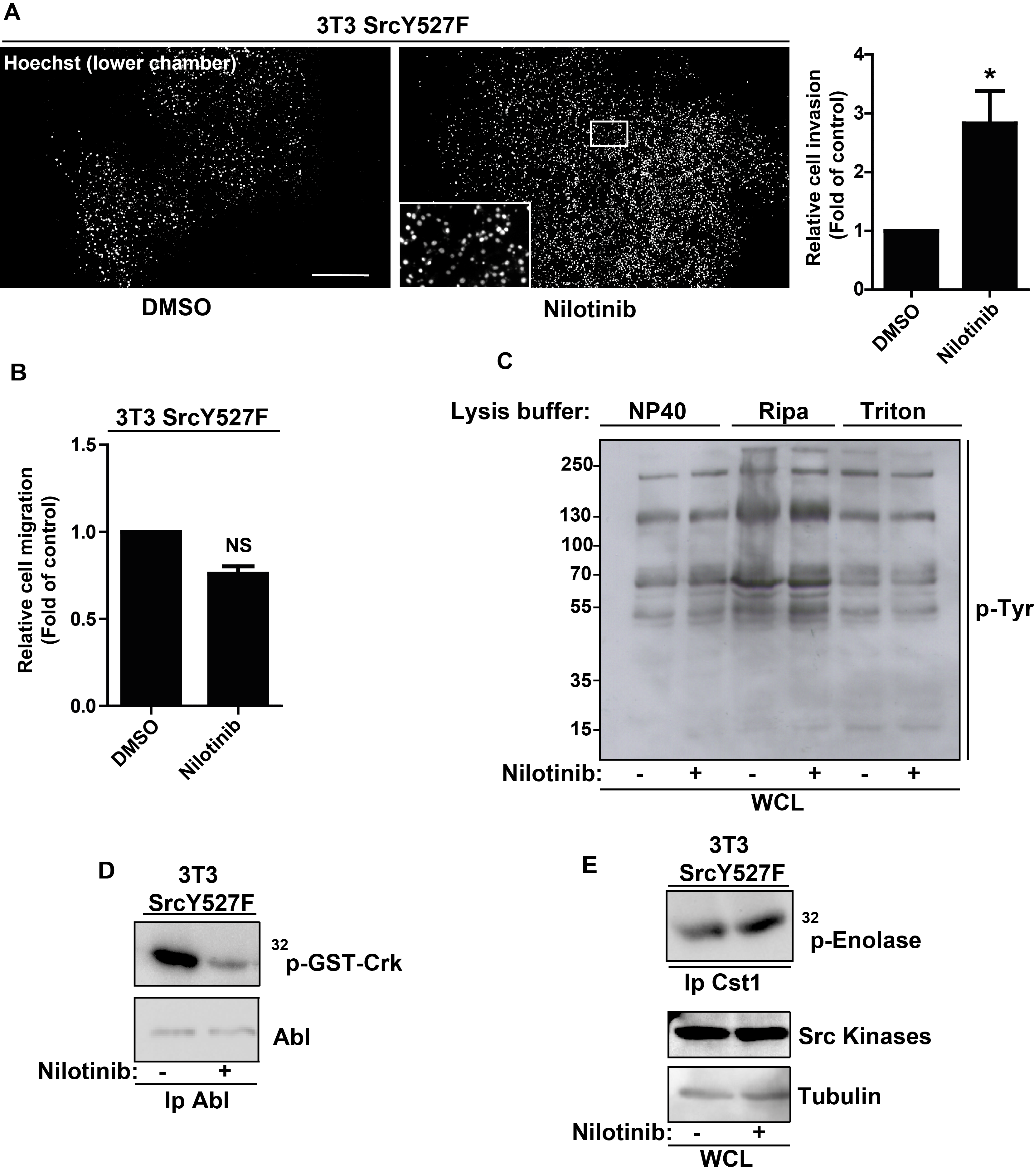

Supplement: S2 Fig — (A, B) Cell invasion and migration studies were done as in Figs. 2 and 3, (C-E) Biochemical analyses were done as described in Figs. 4 and 5. (TIF) [file pone.0118854.s002.tif]

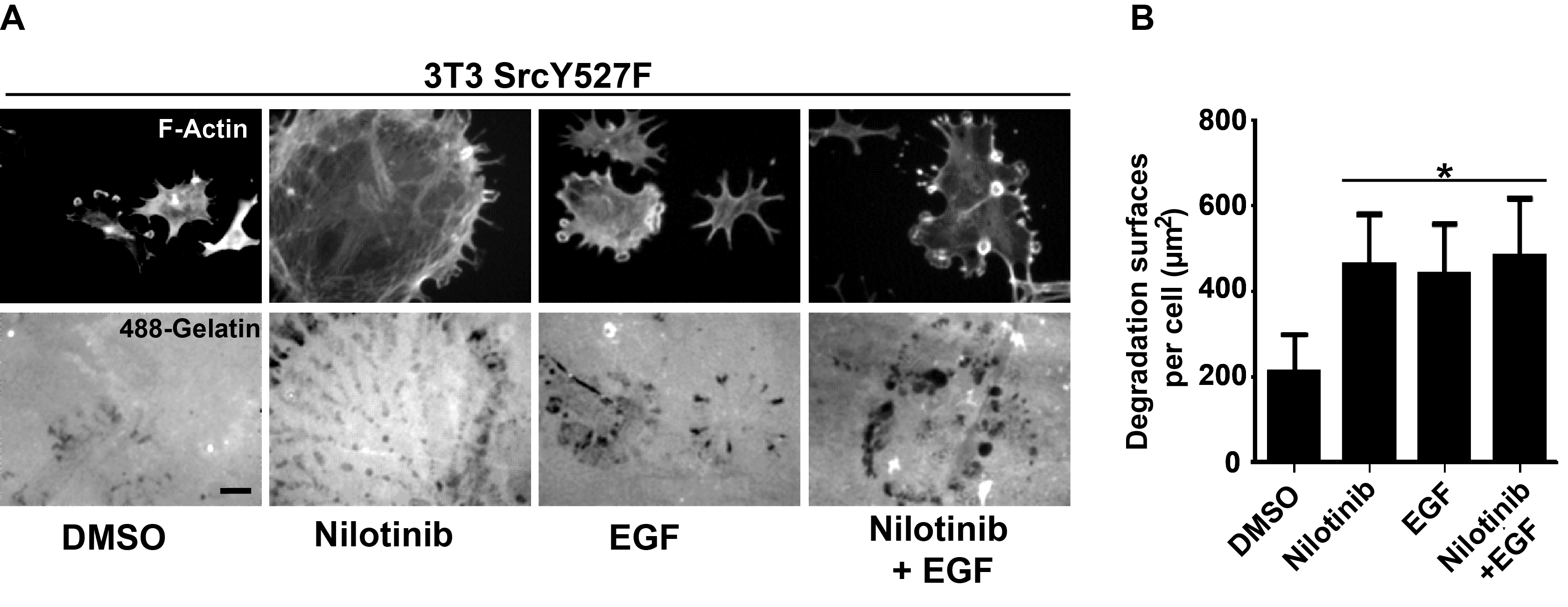

Supplement: S3 Fig — (A, B) 3T3 Src Y527F cells were serum-starved (0.5% serum) overnight, then plated on Oregon Green 488 gelatin and incubated with DMSO, 100nM nilotinib, 200ng/ml EGF or EGF+nilotinb for 3h. After fixation, actin cytoskeleton morphology and gelatin degradation by cells were analyzed as in Fig. 1A. Scale bars: 20μm (TIF) [file pone.0118854.s003.tif]

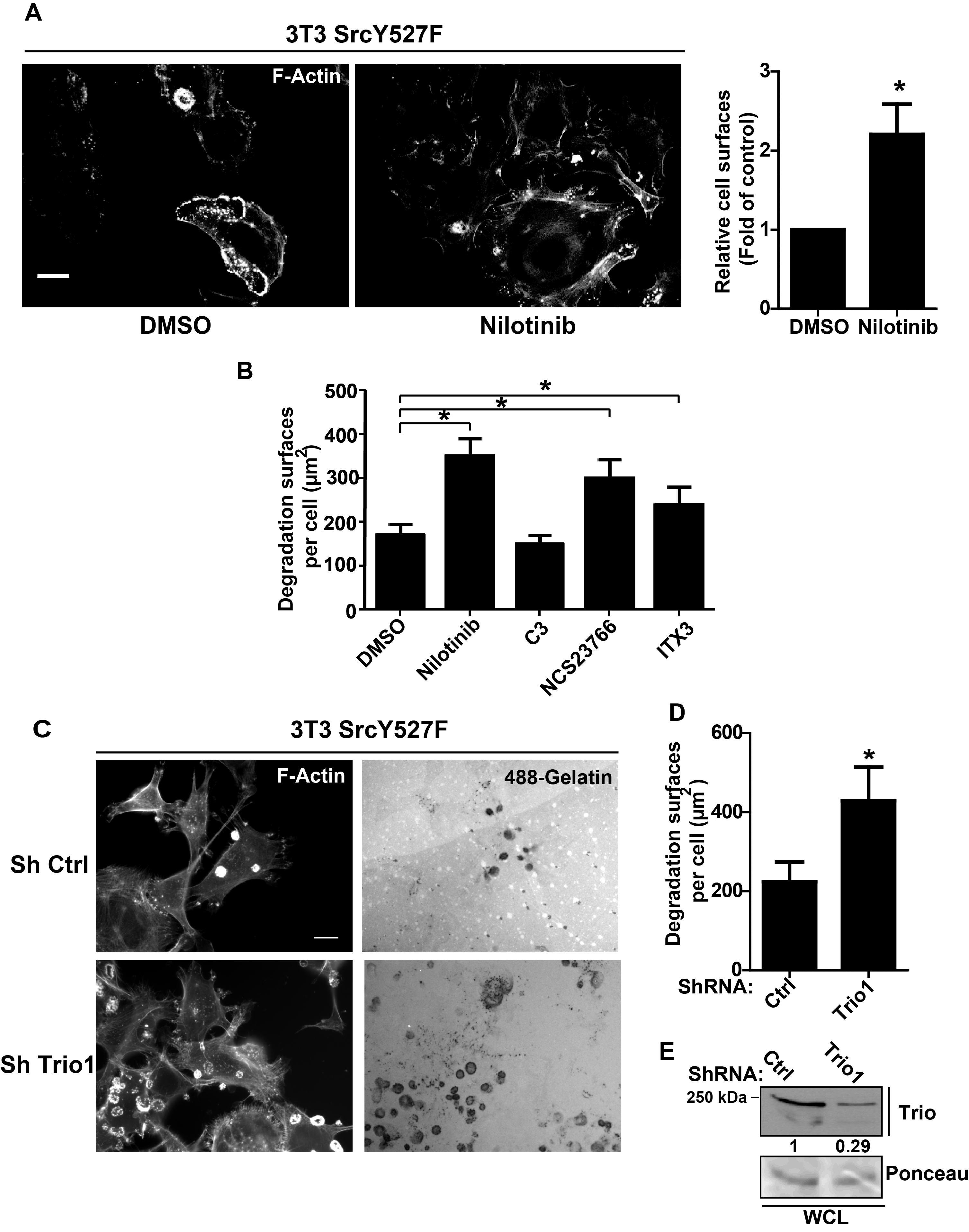

Supplement: S4 Fig — (A) Effect of nilotinib on cell area. The area of 100 3T3 SrcY527F cells plated on Oregon Green 488 gelatin and incubated with DMSO or 100nM nilotinib was measured after F-actin labeling with Alexa 598-phalloidin. Results are the mean ± SEM relative to control. *p<0.05, compared to DMSO-treated cells. (B) The degradation area of 100 3T3 SrcY527F cells plated on Oregon Green 488 gelatin and incubated with DMSO, 100nM nilotinib, 2μg/ml C3, 100μM NSC 23766 or 25μM ITX3 for 3h was measured as described in Fig. 1A. Results are the mean ± SEM, *p<0.05 compared to DMSO treated-cells. (C, D and E) Effects of TRIO down-regulation with shTrio1 in 3T3 SrcY527F cells. (C) Representative examples of actin cytoskeleton morphology and matrix degradation in 3T3 SrcY527F cells infected with control (ShCtrl) or anti-Trio1 shRNAs. Scale bar: 20μm. (D) Quantification of matrix degradation. *p<0.05 compared to ShCtl cells. (E) Western blot showing TRIO expression in 3T3 SrcY527F cells infected with control (ShCtrl) or anti-Trio1 shRNAs. Densitometry quantification normalized to tubulin is shown. (TIF) [file pone.0118854.s004.tif]
